# Healthcare-associated infections in a tunisian university hospital: from analysis to action

**DOI:** 10.11604/pamj.2015.20.197.4062

**Published:** 2015-03-03

**Authors:** Mohamed Mahjoub, Nebiha Bouafia, Waadia Bannour, Tasnim Masmoudi, Rym Bouriga, Radhia Hellali, Asma Ben Cheikh, Olfa Ezzi, Amel Ben Abdeljellil, Njah Mansour

**Affiliations:** 1 Hospital Hygiene Service, University Hospital Centre Farhat Hached, Sousse, Tunisia; 2 Forensic Medical Service, University Hospital Centre Farhat Hached, Sousse Tunisia; 3 Hematology Service, University Hospital Centre Farhat Hached, Sousse, Tunisia

**Keywords:** Healthcare-associated infections, Tunisia, regular prevalence surveys

## Abstract

**Introduction:**

Our study was conducted, in university hospital center (UHC) Farhat Hached of Sousse (city in Tunisian center-east), within healthcare-associated infections (HAI) epidemiological surveillance (ES) program, based, among others, on HAI regular prevalence surveys. Our objectives are to resituate HAI prevalence rate and to identify their risk factors (RF) in order to adjust, in our hospital, prevention programs.

**Methods:**

It is a transversal descriptive study, including all patients who had been hospitalized for at least 48 hours, measuring prevalence of HAI a “given day”, with only one passage by service. Risk factors were determined using Epiinfo 6.0, by uni-varied analysis, then, logistic regression stepwise descending for the variables whose p

**Results:**

The study focused on 312 patients. Infected patients prevalence was 12.5% and that of HAI was 14.5%. Infections on peripheral venous catheter (PVC) dominated (42.2%) among all HAI identified. HAI significant RF were neutropenia (p < 10^−4^) for intrinsic factors, and PVC for extrinsic factors (p = 0,003).

**Conclusion:**

Predominance of infections on PVC should be subject of specific prevention actions, including retro-information strategy, prospective ES, professional practices evaluation and finally training and increasing awareness of health personnel with hygiene measures. Finally, development of a patient safety culture with personnel ensures best adherence to hygiene measures and HAI prevention.

## Introduction

Healthcare-associated infections (HAI) represent a universal public health problem due to their frequency, their seriousness and their additional cost. They increase hospital stay and engender morbidity and mortality with heavy economic and legal burden, everywhere in the world. It is certain that prevention of HAI is well organized in developed countries; however, it is much less in countries with a low socio-economic level who suffer, in majority, from lack of legislations organizing prevention plans in addition to deficiencies of representative data that are essential for monitoring control actions [1, 2].

Epidemiological surveillance (ES) represents a central axe in any HAI prevention strategy. There are different methods of ES, especially prevalence surveys which constitute one of the most common methods adopted, in most countries, allowing follow of HAI’ frequency and epidemiological particularities’ evolution [3]. We have carried out our study, in 2012, at university hospital centre (UHC) Farhat Hached of Sousse, according to HAI ES program based on regular prevalence surveys that have started since 2000 in order to better direct prevention axes. Last investigation is dating back to 2007.

The aims of our study were, in a first time, to estimate HAI prevalence at our hospital and to describe HAI distribution according to anatomical sites, services at risk and germ types; then, in a second time, to identify risk factors associated with HAI occurrence, in order to reorient prevention strategies.

## Methods

We have carried out our study at UHC Farhat Hached of Sousse which has a suburban structure with a medical vocation composed of 26 medical services, 4 surgical services and 9 laboratories. It is equipped with a hospital capacity regarding 698 beds, in 2012. Total staff practicing, at this hospital, is 1661; among them 1354 health professionals with different ranks: 1134 paramedics and 220 doctors. There is an operational hygiene team that defines hygiene policy and formalizes programs that will be adopted then achieved, at hospital. This team works in collaboration with hospital HAI control committee (HAICC). HAI's control and prevention include training, awareness raising, monitoring and assessment of professional practices; and contribute to improvement of quality and safety care.

It is a descriptive transverse survey, conducted in 2012, over a period of 10 days including all patients who had been hospitalized for at least 48 hours, in 16 clinical services of our UHC which are: general surgery, ENT (Ear-nose-throat), ophthalmology, dermatology, hematology, rheumatology, pediatrics, cardiology, medical intensive care, anesthesia-reanimation, pneumology, gynecology (with high-risk and post-operative pregnancies), oncology, psychiatry, internal medicine and infectious diseases, and endocrinology. A single passage has been carried out by service. Criteria of Centers for Disease Control (CDC) Atlanta USA, Prevention National Nosocomial Infection Surveillance (NNIS) and National Healthcare Safety Network (NHSN) system, were used and adapted to our context to define HAI [4].

Study was performed using a questionnaire completed by the investigator in its passage by each service. Finished questionnaires have been daily validated to ensure data completeness. Main sources of data were patients’ medical records, treating physicians and hygiene referents of each service.

Variables measured were related to: patients’ general characteristics, clinical profiles, exposure to invasive devices or a surgical procedure and possible presence of one or several active HAI the day of survey. Data seizure and analysis were carried out anonymously, using software Epiinfo 6.0.

In order to identify HAI risk factors, we have proceeded by a uni-varied analysis comparing patients who have presented at least one HAI to those who do not have, in relation with different variables measured, using chi2 and student tests (t-test). Variables whose p[5].

## Results

### Descriptive results

#### Population characteristics

A total of 312 patients were observed at the day of survey. Patients’ median age was 47 years. 72.7% of patients were hospitalized in medical services (including 2.5% hospitalized in intensive care unit) and 27.2% in surgical services. Diabetes (20.5%) and immunosuppression (20.5%) were main intrinsic risk factors found in patients included in the study, followed by obesity (16.7%). Peripheral venous catheter (PVC) was the most frequently encountered medical device (65.7%) followed by exposure to surgical procedure (15,7%) and urinary probe (9.6%).

Among the 49 operated patients, 17 have received a prophylactic antibiotic treatment, which corresponds to 34.7%. Thus, 10 patients (10/17) received a single antibiotic and 7 (7/17) have received a double antibiotherapy. Predominant prescribed prophylactic antibiotics were association of amoxicillin and clavulanic acid (664.7%), then Nitroimidazoles (41.1%).

Relatively to profile of patients undergoing surgery, 85.7% had an ASA grade of 1 or 2; and 81.6% were operated for a clean or clean-contaminated surgery; and intervention duration was less than 2 hours in 83.7%. In addition, NNIS grade was equal to 0 or 1 in 83.7% of patients; lastly, only 77.66% of the operated were programmed.

#### Characteristics of healthcare-associated infections

Among a total of 312 patients included in this study, 39 have submitted at least one HAI which corresponds to a prevalence rate of 12.5% (CI 95% (0.08 -0.16)). HAI number identified in these patients is 45, meaning a prevalence rate of 14.4% (CI 95% (0.10 - 0.18)). Among these 39 patients with HAI, 22 were hospitalized in medical services (56.4%) and 17 were hospitalized in surgical services (43.66%). Calculation of infected patients’ prevalence rate by service has helped to identify HAI high risk services where HAI prevalence rate varied between 12% and 45%. These services were: hematology, general surgery, medical intensive care unit, gynecology-obstetrics/maternity, ENT and pneumonology.

According to infection site, infections on PVC were predominant (42.2%) followed by respiratory infections (15.6%), then ENT infections (13.4%). Among these 45 HAI detected, 13 microbiological samples have been done representing only 28.8% of the HAI identified; 6 samples were positive (HAI bacteriologically documented), 4 of them to negative Gram Bacilli (NGB).

#### Analytical results

We proceeded with a uni-varied analysis comparing infected patients to those not infected according to their general characteristics and clinical profile, at admission. Among factors related to patient, only neutropenia frequency is revealed significantly higher beyond those having presented a HAI, against those who did not have it (Table 1). As for invasive care exposure, our study showed that PVC exposure frequency was significantly higher in infected patients (Table 2). Subsequent to multivariate analysis, three variables have been proved as independent risk factors of HAI occurrence, as to know: PVC, gastric tube and neutropenia (Table 3).

**Table 1:** Comparison according to general characteristics and clinical profile of patients (N= 312)

General Characteristics and clinical profile at admission	Group 1 Presence of HAI (N= 39)		Group 2 Absence of HAI (N= 273)		p
	N	%	N	%	
**General characteristics**					
Average Age +(+/- SD)	44.8 +/- 21.1 years		44.8 + /- 22.7 years		0.99
Female	26	66.7	151	55.3	0.18
**Admission clinical profile**					
History of hospitalization in the last 12 months	11	28.2	90	33	0.55
Antibiotic therapy in the last 3 months	14	41.2	62	25.5	0.05
Diabetes	8	20.5	56	20.5	1
Obesity	6	15.4	46	16.8	0.81
Immunosuppression	11	28.2	53	19.4	0.20
Neutropenia	7	17.9	5	1.8	<10-4
**Admission characteristics**					
Emergency admission	21	53.8	114	41.8	0.15
Transfer	1	2.6	13	4.7	0.4

Test of Student was used to compare average age between the two groups of patients

**Table 2:** Comparison depending on exposure to invasive care (N= 312)

Invasive Care	Presence of IN (N1 = 39)		Absence of IN (N2 = 273)		p
	N	%	N	%	
Urinary Probe	5	12.8	25	9.2	0.46
Gastric Tube	3	7.7	5	1.8	0.065
PVC	34	87.2	171	62.6	0.003
Intubation/VA	4	10.3	11	4	0.10
Surgical Intervention	10	25.6	39	14.3	0.068

**Table 3:** Independent risk factors of HAI

	Full Model		Final Model	
	OR IC 95%	p	OR IC 95%	p
Female	2.95 (1.14 - 7.63)	0.026	-	
Admission in emergency	2.31 (0.95 - 5.56)	0.062	-	
Antibiotherapy in the last 3 months	1.73 (0.74 - 4.04)	0.20	-	
Immunosuppression	0.58 (0.12 - 2.74)	0.49		
Neutropenia	28.34 (3.91 - 205.43)	0.001	10.28 (3.00 - 35.17)	<10-4
Gastric Tube	3.78 (0.49 - 29.24)	0.20	5.17 (1.13 - 23.68)	0.03
PVC	2.42 (0.83 - 7.02)	0.10	3.48 (1.29 - 9.37)	0,014
Intubation/VA	1.90 (0.35 - 10.35)	0.45		

## Discussion

Prevalence surveys constitute the best monitoring method in several countries, especially when there is lack of mechanisms assuring continuous HAI monitoring [6]. They were even recommended by World Health Organisation for national or international studies, allowing estimation of problem's magnitude and definition of actions’ priority [7]. In fact, this type of survey, has the advantage of being simple and less costly compared to other methods of surveillance. In addition, it has an advantage of providing quick information for an immediate reaction. It, also, able staff to be aware of risks related to HAI. Moreover, distribution of these investigations at a regular interval, allows us to measure secular trends and to assess global impact of prevention policy.

### Discussion of results

Our investigation revealed an infected patients’ prevalence of 12.5% and a HAI prevalence of 14.5%. This figure is at the same range of rates published in literature. In fact, a number of studies, conducted in different countries, found prevalence rates that vary between 3.3% and 19.9%. Indeed, in United Kingdom HAI prevalence has decreased between 1994 et 2006 from 9% to 7,59% [8, 9]. This prevalence, in 1996, was 5.9% in Greece [10] and 3.5% in Germany [11]. In France HAI prevalence has changed between 1996 and 2007 from 6.7% to 3.3% [12–14]. It was in Norveege 6.1% in 1997 [15] and 6,8% in 2005 [16], it was 8% in Denmark in 1999 [17], 4,6% in Slovenia in 2001 [18] and 6,9% in Indonesia in 2002 [19]. HAI prevalence has a regressing tendency in Algeria in 1993, 2001 and 2005 it went respectively from 16,2% [20] to 9,8% then to 4% [21]. In Morocco HAI prevalence was 6,7% in 2005 [22]. In Tunisia, HAI prevalence has regressed between 1999 and 2005 from 14,1% [23] to 6,9% [24] then it has increased and reached 13%, in 2006 [25]. Finally, HAI prevalence was in Senegal 10,9% in 2007 [26].

In developed countries, reported records in literature are, commonly, considerably lower than in our survey. In fact, comparison is difficult because of methodological differences. These disparities concern criteria for HAI definition, mode of data collection, number of infective site investigated, as well as type of hospital activity or service size studied [27, 28]. Quenon clarified that rates comparison could only be worthwhile if similar methodologies have been adopted [29]. Multi-centric study conducted in 27 hospitals in Mediterranean region, in 2010, involving 4634 patients, has recorded a HAI prevalence of 10.5% [6].

At Tunisian national level, HAI prevalence was significantly lower than that found in our study [24]. However, at a regional level, when comparing results with other Tunisian hospitals, we noted different rates. That can be explained by deficiency of national strategy to prevent HAI, and additionally by lack of standardized prevention methodology.

In literature, nature of service activities contributed to HAI occurrence. Indeed, the highest rate of HAI prevalence was noted in intensive care service [6, 11, 18, 24, 30], as our results revealed. Reasons for that could be attributed to diseases severity, patients’ hospitalization lengthiness and more frequent indications of invasive therapeutic or diagnostic acts [6, 31].

Risk of developing a HAI depends on a number of factors widely described in studies conducted everywhere in the world [4]. Regarding to age and gender, same as a study conducted at UHC Hassan II of Fes, our study has not revealed a significant association between HAI occurrence and these factors, despite a female predominance of 66.7% (p= 0.18) [22]. However, investigations results carried out in Turkey [32], Slovenia [18] and in Albania [33] had objected a significant predominance of HAI among men. As well, it is proved that diabetes and obesity promote occurrence of respiratory and surgical site infections [34, 35]. NosoTun study in Tunisia showed a statistically significant association between HAI prevalence and diabetes (p = 0,009), malnutrition (p < 10^−4^), immunosuppression and neutropenia (p24]. According to mainstream studies, immunosuppression has been recognized as a predisposing factor to HAI [13, 34, 36–39]. Other studies, as ours, did not note association between this factor and HAI [40, 41]. This could be attributed to better healthcare quality delivered to these patients since their admission. Nevertheless, presence of patients’ classification bias, due to different diagnostic criteria could not be formally eliminated in such study. In fact, neutropenia which can be regarded as a good indicator of immunosuppression has been identified as significant HAI risk factor in our study (OR= 10.28 (IC95% (3.00 - 35.17)) and p< 10^−4^). According to Lass-Florl, neutropenia [42]. This factor is also linked to HAI bacteraemia [34, 37, 43].

Many extrinsic risk factors may take part in genesis of HAI. Unfortunately, more than half (65.7%) of our patients, were exposed to PVC. Also, results of multi-varied analysis notified that risk of developing a HAI is multiplied by 3.48 when patients are exposed to PVC (OD = 3.48 (1.29 - 9.37)). These results concord with those of a prevalence survey conducted in Morocco [44]. Several other studies have shown that central and peripheral catheters are potential risks factors of HAI occurrence [15, 35, 36, 39, 40, 45, 46]. Frequently, venous catheters devices give often local infections; moreover, central venous catheters are involved in 90% of bacteraemia [46]. Infection on catheter is, in fact, a matter of its installation quality, maintenance care and ablation delay [34, 45–47].

Although, 15.7% of our patients were exposed to a surgical intervention, proportion of operatory site infection (OSI) was low, about 4.4% (2 OSI on 49 surgery); this could be explained by patients clinical profile; more than 80% had a score NNIS equal to 0 or 1. Generally, association between exposure to a surgical intervention and HAI occurrence (regardless of anatomical site) was not regularly established [10, 11, 36–38]. Furthermore, 34.7% of our patients have received a prophylactic antibiotic treatment; the most frequently prescribed antibiotics were association of amoxicillin and clavulanic acid (64.7%) followed by Nitroimidazoles (41.1%), as reported in literature [27]. Association between parenteral feeding and HAI occurrence has been demonstrated in a Mexican study [48]; this association could not be tested in our study because of exposed patients’ few number. Multi-varied analysis have revealed that gastric tube is an independent risk factor of HAI occurrence (OR= 5.17 (IC95% (1.13 - 23.68)); p= 0.03). In this same sense, a study carried out at Fann university hospital has confirmed association between gastric tube and pulmonary HAI’ occurrence [26].

Comparison of HAI distribution according to anatomical site, between countries and even between establishments of the same country, must consider type of institution activities on one hand, and on the other methodology of data collection, such as: adopted definitions, compendium completeness, study period and investigators expertise [49]. Nevertheless, our study revealed a high proportion of vascular infection particularly on PVC about 42.2%, a result far higher than that was reported in literature; indeed, NosoTun investigation in Tunisia revealed only 8.6% of infections on PVC [24]. These infections deemed preventable in more than one third of cases, unless hygiene rules compliance when installing, manipulating and removing PVC. These infections should managed by a specific prevention action strategy. Microbiological documentation has been the main limitation of our study, since that among the 45 HAI found, only 13 samples have been carried out, among them 6 are returned positive, predominantly to NGB. Predominance of NGB in HAI is common to most of studies, despite variability of bacterial species from one country to another [12, 22, 24].

### Prevention strategy

Considering HAI preventive methods, leads us to discuss these infections avoidability and interventions efficacy on their risk factors. As known, approximately one third of HAI is avoidable, regarding results of NNIS survey dating from some thirty years [50]. Besides, HAI prevention should take part of a risk management program including prevention axes which are defined periodically and previously by hospital HAI control committee. Further, establishment of an ES system at hospital, targeting services at risk, is widely recommended. HAI prevention program includes, relating to health-carers: information and awareness, continues training, insisting on action priorities and evaluation of professional practices. These measures are effective when grouped together within the same global strategy recognized under the name of bundle.

## Conclusion

Ultimately, at our hospital, there is a predominance of infections related to PVC, even though they are judged in majority of cases as avoidable HAI. That is why they should be managed with a specific prevention strategy, in which many approaches are included. They are, mainly: retro information of results to each service in order to raise awareness of problem magnitude; prospective ES of infections related to PVC; and finally, evaluation of professional practices in the field of PVC installation, maintenance and withdrawal. Implication of health professionals gets better, by encouraging their commitment and adherence to hygiene measures, as well as, by developing patient safety's culture, which guarantees the best HAI prevention. Elsewhere, prospective studies are desirable in order to describe more accurately incidence as risk factors in each context.
